# Age, Emotion Regulation, and Well-Being after the 2016 Flood

**DOI:** 10.1093/geroni/igab046.3103

**Published:** 2021-12-17

**Authors:** Katie Cherry, Piper Bordes, Matthew Calamia, Emily Elliott

**Affiliations:** 1 Louisiana State University, Louisiana State University/Baton Rouge, Louisiana, United States; 2 Louisiana State University, Louisiana State University, Louisiana, United States; 3 Louisiana State University, Baton Rouge, Louisiana, United States

## Abstract

In 2016, catastrophic flooding in south Louisiana claimed 13 lives with billions of dollars in damage to homes and communities in the decade after Hurricanes Katrina and Rita devastated the US Gulf Coast. In this study, we tested the inoculation hypothesis which predicts that older adults will be less distressed than younger adults due to their prior experience with severe weather events. Participants were 218 predominately middle-aged and older adults who varied in current and prior flood experience: less than half (40%) did not flood in 2016, 31% had flood damage, and 29% had relocated permanently inland after catastrophic losses in the 2005 Hurricanes Katrina and Rita and they flooded again in 2016. Depression symptoms were assessed with the 9-item Patient Health Questionnaire (PHQ-9). Emotion regulation strategies were measured using the Cognitive Emotion Regulation Questionnaire-Short Form. Results indicated that the older adults had fewer symptoms of depression and were less likely to report self blame for flood-related adversities compared to younger adults. The two age groups did not differ significantly on the emotion regulation strategies of acceptance, reappraisal, positive refocusing, other blame, and perseveration. Age was inversely associated with symptoms of depression and the maladaptive strategies of self blame for flood-related misfortune and perseveration over losses. These data support the inoculation hypothesis and suggest that prior severe weather experiences, which are likely for older adults living in hurricane prone areas, are important for post-flood resilience. Implications of these findings for disaster planning and age-sensitive interventions to mitigate adversity are considered.

